# Simultaneous Life Detection and Localization Using a Wideband Chaotic Signal with an Embedded Tone

**DOI:** 10.3390/s16111866

**Published:** 2016-11-06

**Authors:** Li Liu, Chaoyi Guo, Jingxia Li, Hang Xu, Jianguo Zhang, Bingjie Wang

**Affiliations:** 1 Key Laboratory of Advanced Transducers & Intelligent Control System, Ministry of Education and Shanxi Province, Taiyuan University of Technology, Taiyuan 030024, China; guochaoyi0857@link.tyut.edu.cn (C.G.); lijingxia@tyut.edu.cn (J.L.); xuhang@tyut.edu.cn (H.X.); zhangjianguo@tyut.edu.cn (J.Z.); wangbingjie@tyut.edu.cn (B.W.); 2 College of Physics & Optoelectronics, Taiyuan University of Technology, Taiyuan 030024, China

**Keywords:** life detection radar, through-wall, chaotic signal, Colpitts oscillator, split ring, 84.40 Xb, 05.45 Gg

## Abstract

A hybrid life detection radar system which transmits a wideband chaotic signal containing an embedded single-tone is proposed. The chaotic signal is used for target localization by the time-domain correlation method and synthetic aperture technique, and the single-tone signal is used to measure the frequencies of breathing and heartbeat based on an on-chip split-ring integrated sensor and Michelson interference principle. Experimental results in free space and in through-wall scenarios demonstrate that the system can realize human detection and localization simultaneously with high range resolution, high sensitivity, and large dynamic range without complex signal processing. The range resolution is about 10 cm, and the dynamic range is 35 dB for the respiration signal detection and 25 dB for the heartbeat signal detection. Due to its good immunity to interference/jamming and high spectrum efficiency, the proposed system is suitable for post-disaster rescue, elder/infant/patient vitality monitoring, and anti-terrorism enforcement applications.

## 1. Introduction

Non-contact life detection radar sensor has been a growing research interest in recent years, due to its practical applications in anti-terrorism enforcement, rescue scenarios [1,2,3] and remote medicine [4,5,6]. Compared with other vital sign sensors, microwave radar sensors have advantages of high accuracy, high sensitivity, independent of environmental factors, and through-obstacle detection ability.

According to the radar architecture used, there are four typical types: continuous-wave (CW) radar [7,8], frequency-modulated continuous-wave (FMCW) radar [9,10], stepped-frequency continuous-wave (SFCW) radar [11,12] and impulse-radio ultra-wideband (IR-UWB) radar [13,14]. Single-tone continuous wave is also called Doppler radar or interferometry radar, and it has high receiving sensitivity, high measurement accuracy, simple structure, low cost and relatively easy post-processing [15]. However, it is difficult to detect range information, which is important for assisted-living, trapped victim searching, and through wall hostage rescue. Although a frequency-hopping CW radar [16] can provide target localization features, it cannot work well in heavily cluttered environments due to multipath propagation or strong wall reflections. Multiple Doppler radars based on angle of arrival algorithms [17] can estimate the position of moving targets, but they can only determine an enclosed region instead of giving a precise location of the target. The FMCW, SFCW, and IR-UWB radar can localize and separate multiple targets, even in through-wall scenarios, but suffer from higher cost, more complex architecture, and more complicated advanced signal processing algorithms [15,18,19,20,21]. Besides that, to achieve high range resolution a large bandwidth is needed, which will reduce the receiving sensitivity and thus harm the robust detection of the weak vital signal.

Therefore, to overcome the deficiencies of different types of radar system, some hybrid radar systems are presented. Wang et al. [22,23] introduced a hybrid frequency-modulated continuous wave (FMCW) interferometry radar sensor, in which the transmitted signal is a sequence of alternating single-tone signals and linear chirp signals. By incorporating the FMCW mode and the CW interferometry mode, it can detect absolute distance as well as relative displacement motions. Narayanan et al. [24] proposed a portable real-time digital noise radar system, which combines UWB noise radar with Doppler radar using a mode-select switch, and has good ranging, imaging and human micro-Doppler detection capabilities at stand-off distances of up to 9 m and in through-wall scenarios. Later, they introduced a similar hybrid millimeter-wave radar to simultaneously range humans concealed in light foliage and detect the Doppler characterization of human movements [25,26]. These works have demonstrated that hybrid systems containing a wideband signal with a single-tone are also a good choice for life detection radar.

Wideband signals have impulse/short-pulse, linear frequency modulated, step-frequency, or random noise signals. Among these, random signals have attracted increasing attention in many radar fields, including ranging, SAR imaging [27], target tracking [28], ground penetration [29], and through-wall sensing [30]. Since noise signals are featureless and have delta-like autocorrelation functions, random noise radars possess several advantages, such as low probability of detection, low probability of interception, immunity to interference and jamming, and high spectrum efficiency, the last two of which are beneficial for life detection applications. Therefore, some noise or pseudo-noise (PN) life detection radars have been proposed [31,32,33,34]. Sachs et al. [31,32] presented a UWB M-sequence radar for rescue operations in post-disaster scenarios, and later proposed a M-sequence-based UWB sensor network for vitality monitoring of elders at home. Xia et al. [33] proposed a modulated M-sequence UWB life detection radar, which utilizes pulse compression and a linear average to improve the signal-to-noise ratio (SNR), and a hybrid sampling technique to improve the sampling resolution. Susek et al. [34] demonstrated a noise radar with a microwave quadrature correlator receiver, which can precisely and simultaneously detect live human activities like breathing and heartbeats, and their position.

Besides noise and pseudo-random signals, chaotic signals are also random. Compared with random noise, a broadband chaotic signal with a large amplitude can be generated with very simple nonlinear dynamical systems, such as chaotic circuits. Although random signals with comparable bandwidths can certainly be generated by amplifying small-amplitude random sources such as thermal noise sources, it is difficult and costly to have comparable power limited by the broadband large-gain amplifiers [35]. Moreover, unlike noise, chaotic signals are deterministic in that they are controllable and offer the possibility of synchronization [36,37] and security detection. In addition, compared with PN code, chaotic signals have no “code length” limitation which will not render the detection ambiguous. Therefore, chaotic signal has been widely explored in the radar field [35,36,37,38,39,40,41,42,43]. In most existing chaotic radar systems, chaotic signals are generated by a discrete map and used as a baseband signal for modulation, such as frequency-modulation, amplitude-modulation, phase-modulation and pulse modulation [37,38,39,40]. Such chaotic modulation radars have complicated architectures and cannot utilize the broadband properties of chaotic signals. Direct chaotic radars, which transmit a broadband chaotic signal directly, are also presented. Lin et al. [35] proposed a chaotic ranging radar based on an optically injected semiconductor laser, and achieved a range resolution of 9 cm. Zhang et al. [41] exploited a UWB remote ranging radar with 2 cm range resolution, which employs an optical-feedback semiconductor laser with optical injection to generate photonic chaos. Cheng et al. [42] presented a multiple-input-multiple-output chaotic radar, which uses electrical heterodyning to generate uncorrelated multi-channel chaotic waveforms. Jiang et al. [43] suggested a prototype of chaotic radar based on a Colpitts oscillator, and obtained a range resolution of 12.57 cm. We also designed a remote chaotic imaging radar based on optical fiber links, which uses a Colpitts circuit and an external modulation technique on a laser diode to realize remote transmission over a fiber link [44]. Therefore, chaotic signals have been proved to be a promising alternative to noise signals.

In this paper, we propose a novel hybrid life detection radar which utilizes a wideband chaotic signal with a single-tone as the transmitted signal. The chaotic signal is responsible for target localization by a time-domain correlation method and synthetic aperture technique, while the CW signal is used for human detection. The major contribution of this paper is that to our knowledge, it is the first to introduce wideband chaotic signals to the life detection radar field. Although noise signals have been applied for human detection, chaotic signals have better characteristics than noise signals as mentioned before. In addition, different from the existing hybrid life detection radar proposals, the two waveforms are transmitted simultaneously without a switch or some other controller. Due to the featureless properties of the chaotic signal, the two waveforms are independent and will not influence each other. Moreover, a rapid interference phase detection method based on an on-chip microwave sensor is used to detect vital signs, which can make the GHz wideband signal collapse to a single low frequency in the receiver. The sensor with a size of 40 mm × 50 mm is a rectangular split ring integrated with a Schottky diode, and it has high sensitivity and large dynamic range [45]. Therefore, our proposed life detection radar can accurately localize the human beings, and detect their vital signs simultaneously with high range resolution, high sensitivity and without complex signal processing. The paper is organized as follows: Section 2 presents the proposed radar system and its principles, including the characteristics of the composite transmitted signal and the split-ring based sensor, the experimental setup, and the principles of target ranging and human detection. Section 3 illustrates experimental results in free space and in through-wall scenarios. The dynamic range of human detection is also analyzed. Finally, Section 4 offers some conclusions.

## 2. The Radar System and Its Principles

### 2.1. The Transmitted Signal

**Figure 1:**
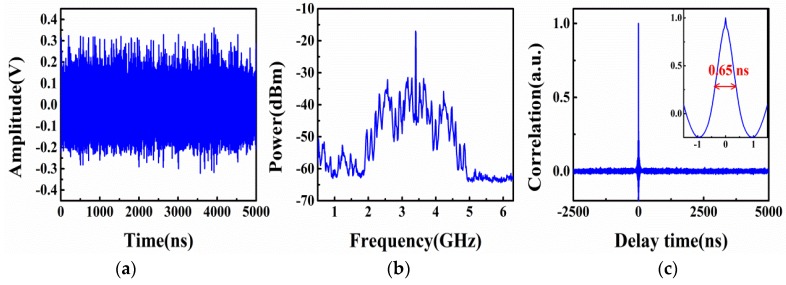
Properties of the transmitted signal. (**a**) Time series; (**b**) Power spectrum; (**c**) Autocorrelation trace.

The transmitted signal consists of a chaotic waveform generated by an improved Colpitts oscillator and a single frequency tone at 3.4 GHz. The use of a chaotic signal generated by a Colpitts oscillator has the following advantages: (1) a wide spectrum is easily achieved without any expensive devices, such as high-speed PN generator, broadband large-gain amplifier, or expensive photoelectric devices needed in the optical generation of chaos; (2) the non-periodic waveform eliminates detection ambiguity which inherently exists in PN radar; (3) the Colpitts circuit is compact and stable, which is beneficial to system integration, and (4) the noise-like waveform ensures excellent jamming resistance. The detailed parameters of the Colpitts circuit can be found in [46]. Figure 1 shows the time series, power spectrum and autocorrelation curve of the transmitted signal. Obviously, the waveform has a noise-like feature and no obvious periodicity, and its peak-to-peak value is approximately 600 mV. The prominent power spectrum spans from 2 GHz to 5 GHz, and the power of the 3.4 GHz is about 20 dBm higher than the average power of the chaotic signal. The autocorrelation trace of the time series shown in Figure 1c exhibits a delta-like profile, and the inset shows the full width at half maximum (FWHM) of the correlation peak is 0.65 ns. According to Wiener-Khinchin theorem, the signal’s autocorrelation function and its power spectrum are Fourier transform pairs, so the FWHM of the autocorrelation function depends primarily on the signal’s bandwidth. While the bandwidth *B* determines the range resolution of the radar system by *c*_0_/2*B*, where *c*_0_ is the speed of light in free space. Then we can deduce that the free-space range resolution equals *c*_0_·FWHM/2 based on −3 dB criterion. Therefore, the theoretical range resolution of the proposed radar system is 9.75 cm.

### 2.2. On-Chip Split-Ring Based Sensor 

**Figure 2:**
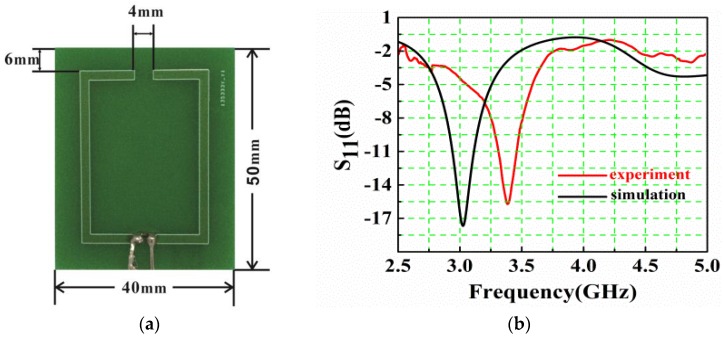
The split-ring based sensor. (**a**) Prototype board; (**b**) Experimental (**red**) and simulated (**black**, without Schottky diode) frequency response curves.

The sensor for vital sign detection is an on-chip solid state sensor which integrates a rectangular split ring operating at a resonance frequency of 3 GHz and a Schottky diode, as shown in Figure 2a. A split-ring sensor has the advantages of high sensitivity, low cost, and easy fabrication, and thus has been successfully applied to be used as biosensors and to detect the dielectric properties of various liquids. The rectangular split ring is firstly fabricated using High Frequency Structure Simulator (HFSS) software and then made into a printed circuit board. It has a copper loop with two symmetric gaps on the FR-4 substrate, and one gap integrates a Schottky diode. Figure 2b shows the experimental and simulated frequency response of the sensor. Note that the experimental one is measured by a vector network analyzer (R&S, ZNB8), and the simulated one obtained by the HFSS software is for the split ring, not the integrated sensor. Clearly, due to the impedance change caused by the Schottky diode, the experimental resonant frequency of the sensor is shifted from 3 GHz to 3.4 GHz, but the shapes of the two curves are in good agreement with each other. Moreover, the sensor acts as a narrow band filter and mainly collects the energy around the frequency of 3.4 GHz, which is one of the main reasons that we can use the composite signal as the transmitted signal.

### 2.3. Experimental Setup

**Figure 3:**
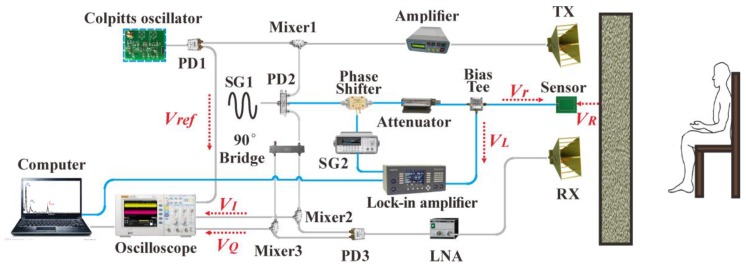
Experimental setup of the proposed life signal detection radar. PD: power divider; LNA: low noise amplifier; SG: signal generator.

Figure 3 illustrates the experimental setup of our proposed hybrid life detection radar, in which devices connected by the gray lines are specific to human target ranging using a chaotic waveform, while devices connected by the blue lines are dedicated to vital sign detection using a continuous wave. The signal composed of the chaotic waveform and the CW waveform is transmitted simultaneously by one antenna, and the echo signal is collected by two antennas (or sensors) for ranging and vital sign detection, respectively.

As for ranging mode, the chaotic signal generated by the Colpitts oscillator is divided into two paths by a power divider (PD1, A-INFO GF-T2-20-3000). One acts as the reference signal, while the other is up-converted by a mixer (MARKI M2-0026), amplified in a maximum 25-dB gain power amplifier (CONQUER KG-RF-10), and transmitted via an 11-dB gain ridge horn antenna (TX, A-INFO LB-10180). The signal collected by another identical ridge horn antenna (RX) is amplified in a low noise amplifier (LNA, MITEQ JSMF4-02K180-32-10P), and then down-converted and demodulated into in-phase/quadrature (I/Q) channels by a power divider (PD3, A-INFO P/N:GF-T3-1000-18000), two mixers (MARKI M2-0026) and a 90° bridge (A-INFO-DQ-T-1018). The I/Q demodulation helps to mitigate the null detection point problem when a very weak or no signal in one of the quadrature channels occurs at certain distances. The demodulated signal *V*_I_(*t*) in I channel, *V*_Q_(*t*) in Q channel and the reference chaotic signal *V_ref_*(*t*) are recorded by an oscilloscope (R&S RTO1024) and then post-processing in the computer. The sampling rate and the bandwidth of the oscilloscope are 5 GSa/s and 2 GHz, respectively.

As for vital sign detection mode, a CW signal at frequency *f* = 3.4 GHz is generated by a signal generator (SG1, AV1487A), and then split by a power divider (PD2, A-INFO P/N: GF-T3-1000-18000). One part of the signal passes through the “reference path”, which includes a voltage-controlled phase shifter (RF-LAMBDA RVPT0204GBC) and an attenuator (Agilent 84968). The phase shifter is controlled by a sawtooth wave at frequency *f_v_* = 100 kHz produced by a signal generator (SG2, Agilent 33220A). The reflected signal, which is modulated by the human chest’s periodic displacement caused by heartbeat and respiration, is received by the sensor with a resonance frequency *f*. The reference signal and the reflected signal couple or interfere at the sensor. Then the interference signal passes through a bias tee (ZFBT-6G-FT), which separates the RF signal and low frequency signal. The resultant low frequency signal is sent to a digital lock-in amplifier (Signal Recovery 7270) for phase measurement, which triggered by the synchronized square wave from the SG2 at a frequency of *f_v_*. The output of the lock-in amplifier is transferred to the computer to process and display. Here, by using the lock-in amplifier, the very weak low frequency signal can be detected from an extremely noisy environment, such as earthquake rescue scene.

### 2.4. Principles

As for ranging mode, target range information can be obtained by correlating the received signal with the reference. Since the autocorrelation of chaotic signal is a delta-like function, the correlation can be expressed as:

(1) $$\sqrt{{[V_{I}(t)⊗V_{ref}(t)]}^{2}+{\left[V_{Q}\left(t\right)⊗V_{ref}\left(t\right)\right]}^{2}}\approx \delta \left(t-\tau \right)$$

where ⊗ denotes the correlation operator, and *τ* is the time delay between *V_ref_*(*t*) and *I* or *Q* output, which includes system delay and round-trip propagation delay from the antenna location to the target. In the experiment, we locate two antennas oppositely and closely to estimate the system delay, and set it as the starting point of the correlation curve. Thus, the position of the correlation peak corresponds to the range of the target. Further, we use synthetic aperture processing and back-projection imaging algorithm to obtain two-dimensional imaging of the target.

As for vital sign detection mode, denoting the amplitude of the reference signal and the reflected signal as *e_r_* and *e_R_*, respectively, the initial phase of the reference path as *ϕ*_0_, the phase difference between the incident and the reflected microwaves due to the oscillation of the target as Δ*ϕ*, then the reference signal and the reflected signal can be written as:

(2) $$\begin{array}{l} V_{r}=e_{r}\cos\left(2\pi ft+2\pi f_{v}t+\varphi_{0}\right)\\ V_{R}=e_{R}\cos\left[2\pi ft+\Delta \phi \left(t\right)\right]=e_{R}\cos\left[2\pi ft+\frac{4\pi d_{0}}{\lambda }+\frac{4\pi x\left(t\right)}{\lambda }\right] \end{array}$$

where *λ* is the wavelength, *d*_0_ is the distance between the sensor and the target, *x*(*t*) is the chest wall displacement caused by heartbeat and respiration. According to the Michelson interference principle [47], the voltage across the sensor due to the wave mixing of two signals is given by:

(3) $$\begin{array}{ll} V\left(t\right) & \propto e_{R}\cos\left[2\pi ft+\Delta \phi \left(t\right)\right]\cdot e_{r}\cos\left[2\pi ft+2\pi f_{v}t+\phi_{0}\right]\\ & =\frac{e_{R}e_{r}}{2}\left[\cos\left(2\pi ft+2\pi f_{v}t+\Delta \phi \left(t\right)+\phi_{0}\right)+\cos\left(2\pi f_{v}t+\phi_{0}-\Delta \phi \left(t\right)\right)\right] \end{array}$$

The above equation includes a second harmonic term with a frequency of 2*f* (which is undetectable using conventional instruments and can be ignored), and a low frequency signal, which carries the phase information caused by the chest wall movement. Therefore, by using the phase shifter and the sensor, the GHz microwave signal is down-converted to a low-frequency signal, which facilitates the signal detection. The phase measurement by the lock-in amplifier corresponds to the phase difference between the reference and the reflected wave. Hence we can simply obtain the frequencies of heartbeat and respiration by performing a Fast Fourier Transform (FFT) on the phase data.

## 3. Experimental Results

Human detection and ranging experiments are performed in free space and in through-wall scenarios. Note that for ranging mode, to enhance the range performance, average method and background subtraction technique are employed to process the correlation function. In our experiments, the correlation is calculated with a length of 100 μs and averaged with 20 measurements. For vital sign detection mode, due to the rapid processing of the lock-in amplifier and the computer, the heartbeat and respiration frequencies can be obtained in real time. A commercial shiatsu heartbeat tester is also used in our experiment to verify the correctness of the results.

### 3.1. Human Detection and Ranging in Free Space

**Figure 4:**
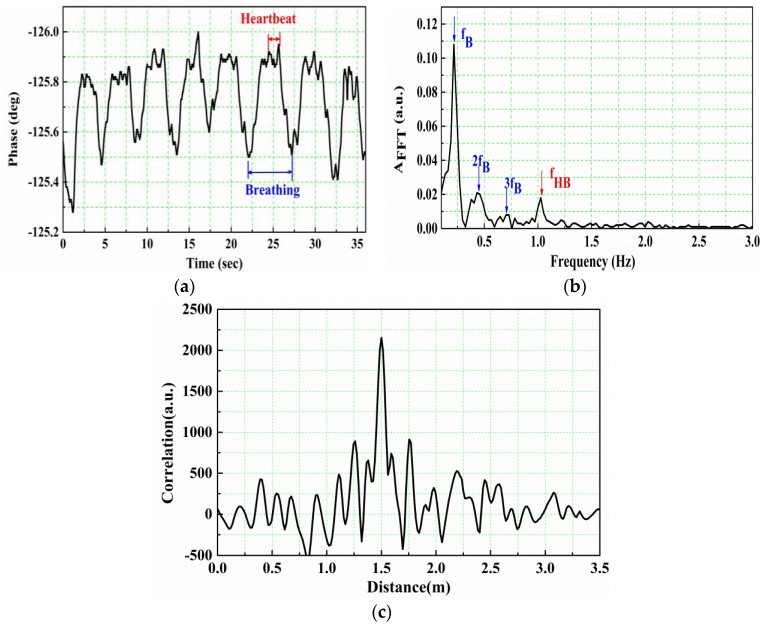
Experimental results of the target at the 1.5 m away from the antennas. (**a**) Raw phase data; (**b**) The FFT amplitude as a function of frequency obtained by performing FFT on the raw phase data; (**c**) Ranging result.

A young male with steady breathing is seated to face the antenna set (composed of one transmit antenna and two receive antenna/sensor) placed 1.5 m away. Figure 4 shows the phase oscillation with time collected by the lock-in amplifier. It can be seen that the heartbeat signal superimposes on the respiration signal, and the respiration signal is stronger than the heartbeat signal. To obtain the frequencies of heartbeat and respiration, a FFT transform is performed on the raw phase data. Figure 4b depicts the result with three significant peaks. The first sharp peak at 0.216 Hz corresponds to the breathing frequency (*f_B_*), the second peak at 0.432 Hz stands for the second harmonic of breathing (2*f_B_*), and the last peak at 1.026 Hz represents the heartbeat frequency (*f_HB_*). There is also another peak corresponds to the third harmonic of respiration (3*f_B_* shown in Figure 4b). Sometimes its height is comparable to that of the heartbeat frequency, but it can be easily distinguished because it is three times the breathing frequency. Meanwhile, the measured beats per minute (BPM) value in the shiatsu heartbeat tester is 62, and then the heartbeat frequency is 1.03 Hz, which is nearly consistent with our experimental result. Furthermore, the amplitude of chest oscillation can be estimated from the maximum and minimum value of the phase data:

(4) $$A=\frac{\left[\phi {\left(t\right)}_{\mathrm{Lmax}}-\phi {\left(t\right)}_{\mathrm{Lmin}}\right]\lambda }{4\pi }$$

where *φ*(*t*)_L_ is the phase measured by the lock-in amplifier. In our experiment, the measured amplitude is about 2 mm–3.5 mm, in good agreement with the results, demonstrated by Droitcour et al. [48], that the Root Mean Square (RMS) for respiration motion is 2 mm. Figure 4c shows the ranging result. From the peak of the correlation curve, we can find that the target location is 1.5 m, which exactly equals to the real value, although the side-lobe level is somewhat high because of the oscillation of the signal’s frequency spectrum.

**Figure 5:**
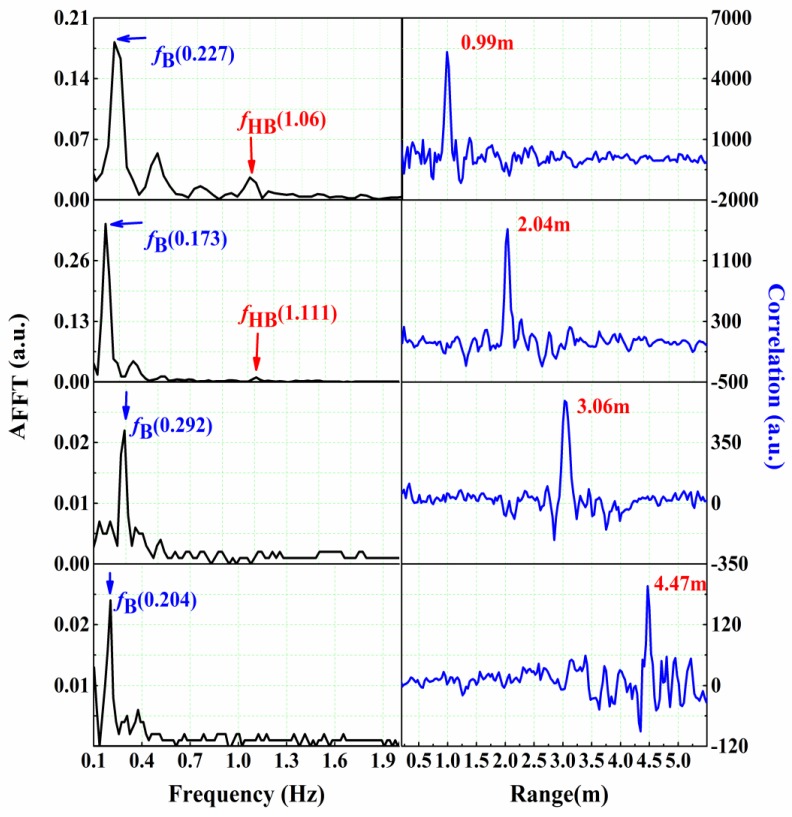
Experimental results in free space for human located at 1, 2, 3, 4.5 m.

Figure 5 shows the human detection and ranging results, when the subject is located at 1, 2, 3, 4.5 m, respectively. Clearly, range information can be obtained within a reasonable error range. As the range increases, the amplitude of the heartbeat frequency decreases, especially when the range is larger than 2 m, it cannot be distinguished. However, the respiration frequency is still distinct. In fact, for rescue applications, the breathing signal is enough for human detection. The largest experimental distance allowed in our laboratory is 6.5 m, where the respiration signal can also be detected, but we cannot get the target location accurately by simple signal processing.

### 3.2. Human Detection and Ranging in Through-Wall Scenarios

**Figure 6:**
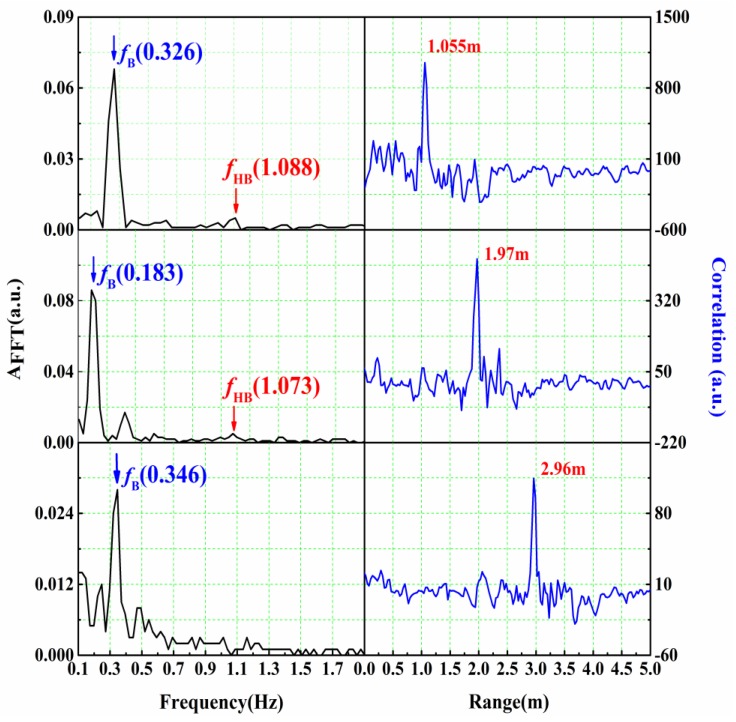
Experimental results with the cinder block wall for a human located at 1, 2, 3 m.

In through-wall scenarios, a 2 m × 3 m (height × width) cinder block wall with 20 cm thick and a mean attenuation of 25 dB at 3.4 GHz was constructed. The antenna set is fixed on the platform at a height of 1.1 m and close to the front wall. A young male with steady breathing is seated at 1, 2 and 3 m, respectively, behind the wall. The results are shown in Figure 6.

Obviously, due to the serious attenuation through the cinder brick wall, the correlation peak is lower and the heartbeat signal is weaker than that in free space. However, when the distance is extended to 3 m, the location of the target can still be detected, and the respiration rate can be identified by the peak in the frequency spectrum. It is demonstrated that the system has good performance in penetrating walls.

**Figure 7:**
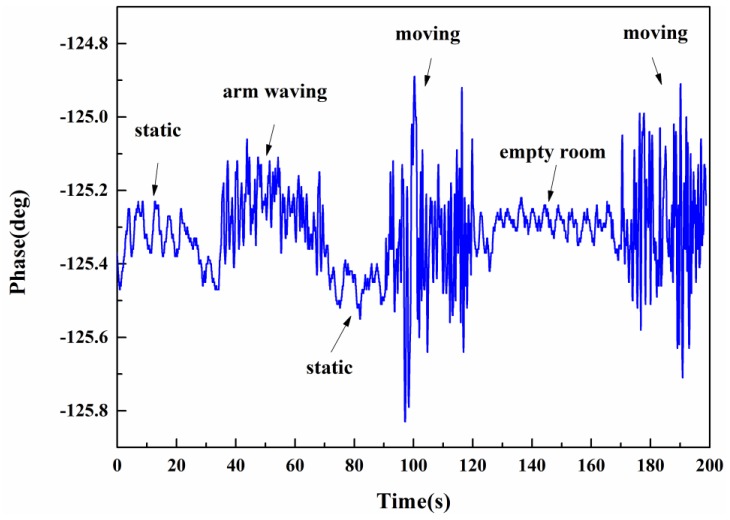
Vital sign signals when a human subject performs different actions (standing still, arm waving, moving, leaving the room) in through-wall scenarios.

Figure 7 illustrates the raw phase data collected by the lock-in amplifier when a young male performs different activities. The subject does the following actions in turn: standing still with steady breathing at 1 m away from the wall, waving the arms without moving, standing still again, moving back and forth, leaving the room, and moving again. Clearly, when the subject is standing still between 0 s and 35 s, the signal presents smaller and periodic fluctuations (around 0.2°), corresponding to the breathing activity. When the subject is waving the arms in position between 35 s and 65 s, the amplitude of the oscillation increases irregularly (around 0.35°). The most intense fluctuation occurs when the subject is walking back and forth (around 1°). Specifically, when the subject leaves the room between 130 s and 170 s, the signal variability is relatively modest (around 0.1°, determined by the system noise level). Therefore, from the signal features, the system has the possibility to detect humans and distinguish different situations, such as no human, static subjects, and moving subjects.

**Figure 8:**
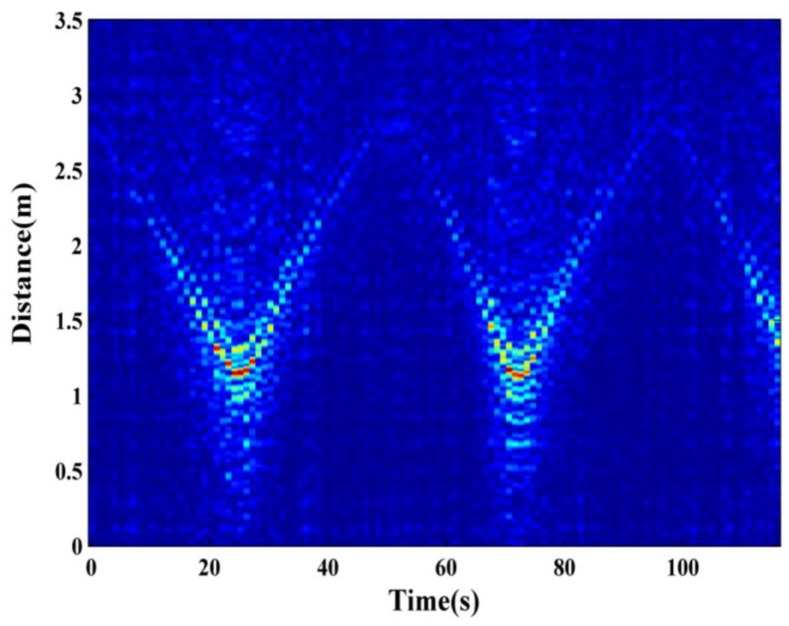
Tracking results of moving subject.

In addition, the proposed radar system can track the moving target. A young male walked uniformly forth and back between 3.0 m and 1.0 m away from the wall. Figure 8 illustrates the result. We can find that although many artifacts exist, the movement route of the subject can be obtained, and the power of the signal decreases as the distance increases. Note that here we just use back ground subtraction method to process the raw data.

**Figure 9:**
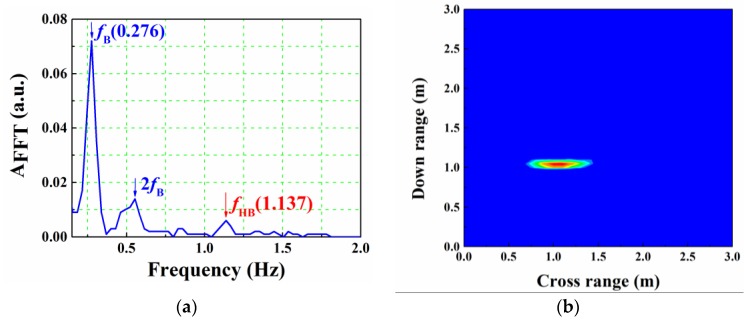
Human detection and imaging result for human located at (1.05, 1.05) m. (**a**) Vital sign detection result; (**b**) Two-dimensional imaging result.

By using the synthetic aperture technique, we can get two-dimensional imaging results. The imaging region is 3 m × 3 m, and a young male with steady breathing is located at (1.05, 1.05) m. The antenna set is moved along the wall with a step size of 5 cm. Thus, there are 61 groups of data, and then these data are used for correlation calculation to obtain range information. After using background subtraction method, back-projection algorithm and threshold method, two-dimensional image is obtained. Meanwhile, vital sign detection is real-time measured by the lock-in amplifier during the scanning, and the phase data with the largest reflection is selected. Figure 9 depicts the human detection and imaging results. Obviously, breathing and heartbeat frequencies are distinct, and the location of the target is close to the real value.

**Figure 10:**
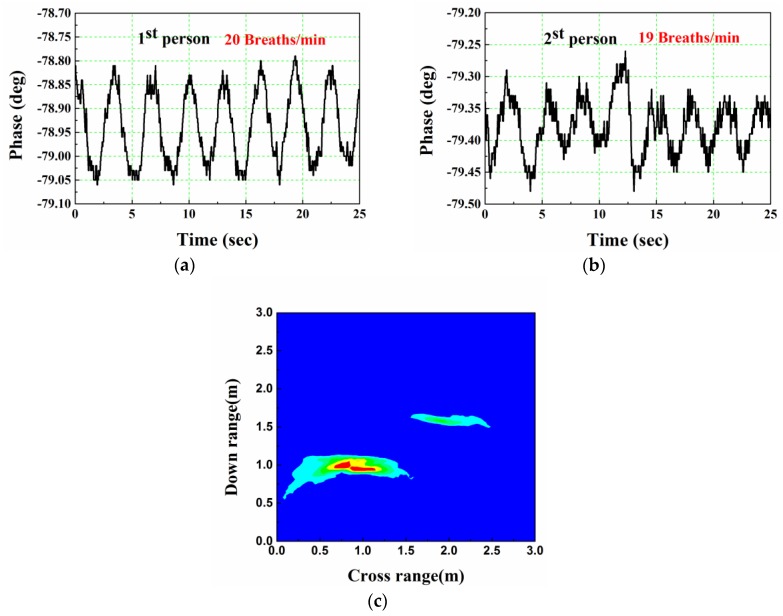
Human detection and imaging result for two human located at (0.8, 1.0) m and (2.0, 1.5) m, respectively. (**a**) Vital sign data of 1st human; (**b**) Vital sign data of 2nd human; (**c**) Two-dimensional imaging result.

Figure 10 shows the experimental result for two humans located at (0.8, 1.0) m and (2.0, 1.5) m, respectively. Obviously, targets can also be easily identified. However, some ghost images exist, due to the interferences and multiple reflections between the targets. The phase data in Figure 8b,c show that two subjects have a similar respiration rate of around 20 breaths/min (obtained by FFT operation). For the second human, who are farther away from the wall, the signal’s SNR is lower than that of the first human, and much less than that in free space, shown in Figure 4c.

### 3.3. The Dynamic Range Analysis

**Figure 11:**
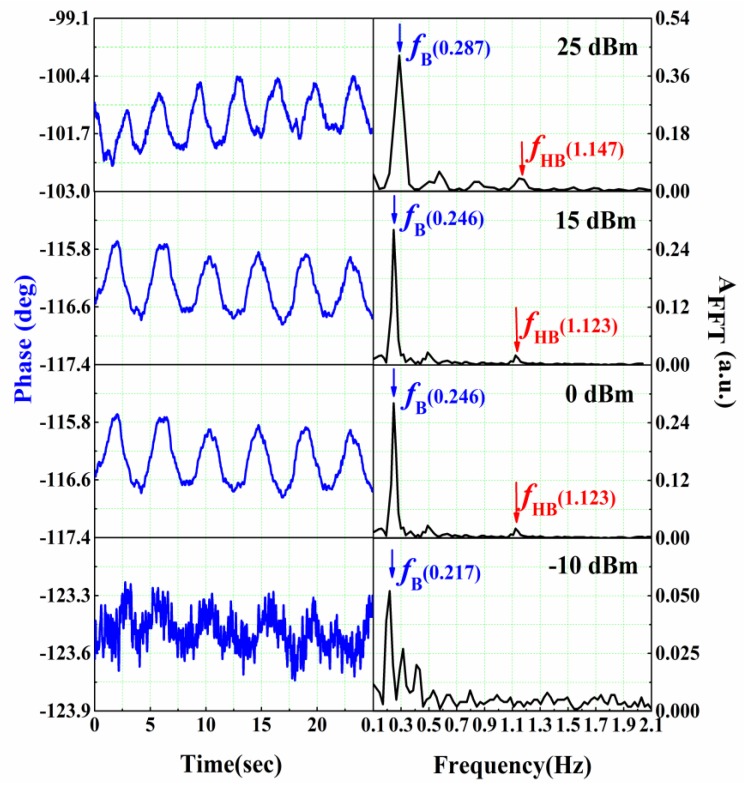
Phase data of the vital sign signals and the corresponding FFT results for different powers.

In our proposed system, the vital sign detection is more sensitive than ranging detection. Here, the power dependence of the detected respiration and heartbeat signal in free space is analyzed. The subject is located at 1 m away from the antenna set. Figure 11 illustrates the phase data of the vital sign signal and the corresponding FFT results for different transmit powers.

Clearly, the noise level increases as the power decreases, but the respiration signal can be seen from 25 dBm to −10 dBm power, while the heartbeat signal cannot be found when the power is less than or equal to −10 dBm due to the low SNR. Therefore, the dynamic range of our present set-up, is about 35 dB for the respiration signal detection and the 25 dB for the heartbeat. Using the inverse square law of the radiation, we estimated that, ideally, at 25 dBm microwave power, we can detect the respiration frequency even if the target is 56 m away from the system, and the heartbeat is 18 m.

## 4. Conclusions

This paper has developed a hybrid life detection radar system which uses a wideband chaotic signal with an embedded single-tone. The composite signal is transmitted simultaneously, and the received signal is collected by a wideband horn antenna and an on-chip split-ring integrated sensor, respectively. The system can detect the breathing signals, heartbeat signals, and localize the target simultaneously. It has high range resolution, about 10 cm, due to the delta-like autocorrelation function of the transmitted signal, and high sensitivity for vital sign detection due to the integrated sensor. Meanwhile, by using the phase shifter and the sensor, the GHz wideband signal collapses to a single low frequency in the receiver, which can simplify the detection method and post-processing, and by using the digital lock-in amplifier, real-time vital sign detection can be achieved. The system also has the ability of discriminating different situations, tracking moving targets, and detecting multiple targets in through-wall scenarios. Moreover, the system has relatively large dynamic range, about 35 dB for the respiration signal detection and 25 dB for the heartbeat signal detection, which is beneficial in strong clutter environments. Therefore, the proposed system can be applied in rescue applications, such as searching for trapped people during a fire or earthquake, and elder/infant/patient vitality monitoring in the hospital or at home. Furthermore, due to the featureless nature of the transmitted signal, several radar systems can share the frequency spectrum, and work together with slight cross-interference to construct a radar network, which is favorable in practical applications.
